# The more you automate, the less you see: Hidden pitfalls of autonomous AI scientists

**DOI:** 10.1073/pnas.2610214123

**Published:** 2026-10-05

**Authors:** Ziming Luo, Atoosa Kasirzadeh, Nihar B. Shah

**Affiliations:** ^a^ Machine Learning Department, School of Computer Science, Carnegie Mellon University, Pittsburgh, PA 15213; ^b^ Department of Philosophy, Dietrich College of Humanities and Social Sciences, Carnegie Mellon University, Pittsburgh, PA 15213; ^c^ Computer Science Department, School of Computer Science, Carnegie Mellon University, Pittsburgh, PA 15213

**Keywords:** AI, AI for science, AI scientist system, responsible AI

## Abstract

The rapid rise of autonomous AI scientists marks a paradigm shift in scientific discovery by automating the research lifecycle. Yet their rushed development has outpaced critical oversight, leaving key workflow decisions dangerously unscrutinized. We present a much-needed systematic analysis of open-source AI scientist systems, investigating four primary pitfalls: inappropriate benchmark selection, data leakage, metric misuse, and post hoc selection bias. Through controlled experiments that isolate each pitfall, we find systematic vulnerabilities across two representative open-source systems. Crucially, we find that these flaws are largely invisible at the level of the final manuscript, suggesting that current manuscript-centric peer review paradigms are fundamentally insufficient for ensuring the integrity of automated research. We further propose mitigation strategies and demonstrate that access to full workflow artifacts (log traces and code) enables more effective auditing. Our findings suggest that journals, conferences, and researchers should move beyond manuscript-only evaluation toward process auditing the end-to-end workflow artifacts of AI scientist systems.

## Significance

AI systems are increasingly capable of autonomously conducting scientific research, from generating hypotheses to writing full papers. However, our study finds that these “autonomous AI scientists” can introduce systematic methodological pitfalls such as biased evaluation, undisclosed data manipulation, and selective reporting that are not visible in the final manuscript. As a result, their outputs may appear rigorous while concealing critical flaws. We find that manuscript-based evaluation alone is insufficient to detect these methodological issues, whereas access to the full research workflow artifacts greatly improves reliability. These findings thus motivate stronger transparency requirements for accountable AI-driven research.

The rapid rise of AI scientists—systems that automate the entire research lifecycle from hypothesis generation to research paper production—signals a paradigm shift in scientific discovery (1). By integrating large language models (LLMs) with simulation environments, automated planning, and evaluation pipelines, these systems can execute end-to-end scientific investigations with minimal human oversight, promising to accelerate discovery, reduce costs, and broaden access to research. The AI Scientist v2 (2) recently reached a critical milestone when its autonomously generated research paper was accepted at a workshop of the International Conference on Learning Representations 2025. This momentum is accelerating: Zochi (3) recently secured an AI-generated paper acceptance at the ACL 2025 main conference, one of the most prestigious venues in computational linguistics. Beyond individual papers, the broader feasibility of AI-driven research was recently tested at scale during the Agents4Science workshop, which explored the end-to-end use of AI scientists as both authors and reviewers (4). These achievements highlight a growing consensus that autonomous systems are no longer merely assistive tools but are increasingly capable of driving independent, high-impact scientific discovery.

Yet, the enthusiasm surrounding AI scientists has outpaced the development of oversight and accountability mechanisms, resulting in a transparency gap that leaves their internal logic dangerously unscrutinized. Left unchecked, these systems risk polluting the scientific record with flawed or fabricated findings, an epistemic threat amplified by the unprecedented speed of automated production. Beyond the widely discussed risks of hallucinations and jailbreaking (5, 6), we observe that AI scientists’ automated pipelines are harboring more insidious failures: methodological flaws that evade easy detection. Safeguarding scientific integrity—such as the rigor of experimental design, the transparency of methodological steps, and the reproducibility of findings—becomes a critical and urgent challenge.

In this paper, we present a systematic investigation into whether current AI scientist systems consistently adhere to the established norms of scientific practice. In doing that, we examine four critical methodological pitfalls in machine learning research systems: 1) Inappropriate benchmark selection: the cherry-picking of favorable benchmarks; 2) Data leakage: the presence of data leakage between training and evaluation; 3) Metric misuse: the misuse of metrics to distort method effectiveness; and 4) Post hoc selection bias: a practice akin to p-hacking where only positive results are reported. These pitfalls are not unique to AI systems and have long been observed in human scientific practice; however, autonomous AI scientist systems may reproduce or amplify such methodological problems at scale under optimization pressures. While we focus on AI scientists in machine learning research as a primary testbed for autonomous agents, our results provide critical insights for the transition toward automated discovery in all experimental science disciplines.

Diagnosing these failures presents significant empirical challenges. First, the pervasive nature of web-scale pretraining makes data contamination nearly inevitable when using existing datasets. This threatens the validity of the evaluation because an AI scientist system’s apparent performance can be due to memorization instead of genuine inference. Second, task design must be sufficiently granular to isolate specific failure modes. For instance, an evaluation of metric misuse must be conducted under a task that is amenable to multiple suitable metrics. Third, the experimental conditions should isolate each specific failure mode, controlling for confounding factors. For instance, when investigating whether an AI scientist system chooses easier benchmarks, we need to distinguish the tendency for agents to select commonly used benchmarks rather than the most appropriate ones. To overcome these hurdles, we develop an experimental environment featuring a fully controlled task that exists, to the best of our knowledge, outside the scope of internet-scale corpora. This environment allows us to isolate failure modes by constructing independent experimental conditions, randomizing system inputs to mitigate potential confounders, and auditing internal decision-making traces to pinpoint exactly when and how methodological failures occur. Under this experimental design, we evaluate two of the most prominent open-source AI scientist systems—Agent Laboratory (7) and The AI Scientist v2 (2). Both are LLM-agent-based machine learning research systems that represent the frontier of contemporary automated research pipelines. They are designed to manage the scientific workflow end to end, from hypothesis generation and experiment execution to paper writing, with minimal or no human intervention.

Our experiments show that existing AI scientists harbor syste-matic failure modes that propagate silently throughout the research lifecycle. We find that, first, rather than selecting benchmarks that rigorously evaluate their proposed methods, these agents preferentially default to well-established state-of-the-art (SOTA) benchmarks or make arbitrary selections driven by presentation order. Second, we find no evidence of explicit peeking into held-out test data. However, both systems exhibit a highly concerning tendency to generate synthetic datasets that are never documented in the manuscripts they generate, or silently subsample provided data during experimentation. These practices can inflate reported performance while rendering the results unverifiable or irreproducible. Third, we do not find evidence of intentional metric manipulation in either system. However, metric selection is sensitive to prompt framing and shows weak adherence to evaluation specifications. In particular, Agent Laboratory’s selection is influenced by the metric order in the prompt, while The AI Scientist v2 more frequently substitutes or replaces the intended metrics. These behaviors indicate instability rather than deliberate selective reporting. Finally and critically, we uncover a structural post hoc selection bias embedded in the agents’ internal reward mechanisms. These systems decide which experiments to report using rewards that directly observe performance on the held-out data, preferentially elevating results that perform well on final evaluation targets, even when earlier evidence of robustness or generalization is weak. This behavior is functionally equivalent to automated p-hacking for surfacing false positives while suppressing negative or inconclusive evidence.

For centuries, scientific accountability has centered on the written paper as the primary item for review and validation (8, 9). However, in the era of autonomous discovery, our analysis reveals systematic pitfalls in Al scientist systems that warrant careful reexamination of this manuscript-centric oversight model. To assess these risks, we developed an easily implementable LLM-based auditing framework to probe for methodological pitfalls in the outputs of AI scientist systems. We also constructed a balanced dataset of positive and negative examples for each pitfall category, where positive examples include both naturally occurring failures observed in system outputs and induced failures intentionally curated by controlling perturbations of the workflow—especially to cover rare or absent cases in our experiments. When testing our LLM-based auditor on this dataset, we find that auditing final manuscripts alone yields poor performance barely better than chance (binary classification accuracy 53.7%, F1 score 0.42). This failure arises because autonomous agents, programmed to optimize for “publishable” results, naturally generate the polished narratives and selectively report results, while omitting the intermediate decisions, discarded experiments, and undocumented data manipulations that shape those outcomes. As a result, many methodological failures are effectively invisible at the level of the published paper. In contrast, when our LLM-based auditor is instead granted access to full execution records—including time-stamped decision logs, intermediate outputs, and generated code that document each step of the research process—detection accuracy improves substantially (81.5% accuracy, F1 score 0.81). These results expose a fundamental misalignment between the opacity of autonomous research agents and the historical assumptions of manuscript-centric peer review. We therefore argue that safeguarding the integrity of AI-generated science will require the development of appropriate scientific norms: developers should proactively audit their systems for the pitfalls identified here and maintain comprehensive log traces for every stage of the workflow, while journals and conferences must move beyond evaluating static papers and mandate the submission of complete research logs for autonomously conducted studies. Without such transparency, the accelerating deployment of AI scientists risks scaling not only discovery but also systematic methodological failure.

## Related Work

Automating the scientific enterprise has been an explicit research goal of computational and AI researchers for several decades (10–12). For instance, King et al. (10) demonstrated the feasibility of fully automated systems for generating and testing scientific hypotheses, exemplified by the “Robot Scientist” capable of independently identifying functional genes in yeast metabolism. Sparkes et al. (11) further advanced this line of work by integrating automated reasoning and laboratory robotics, aiming for autonomous scientific discovery with minimal human intervention. The advent of LLMs, along with their multimodal variants and agent-based extensions, has led to a surge in the development of automated scientist systems. We summarize several representative SOTA systems in Table 1.

**Table 1.** LLM-based AI scientist systems

System Name	HG	EE	PW	PR	Discipline	Open-Sourced
The AI Scientist v1 (13)	✓	✓	✓	✓	Computer science	Yes
Agent Laboratory (7)	✓	✓	✓	×	Computer science	Yes
Carl (14)	–	–	–	×	Computer science	No
The AI Scientist v2 (2)	✓	✓	✓	×	Computer science	Yes
Zochi (3)	–	–	–	×	Computer science	No
Robin (16)	✓	–	×	×	Biomedicine	Yes
NovelSeek (15)	–	✓	×	×	Multiple disciplines	No

Referring to ref. 1, the workflow of the AI scientist systems is divided into four stages: HG = Hypothesis Generation, EE = Experiment Execution, PW = Paper Writing, PR = Peer Review. The order of AI scientist systems in the table reflects the time of their first appearance. “✓” indicates fully automated, “–” indicates semiautomated with human feedback and “×” indicates not supported.

There are different types of AI scientist systems, including those that are claimed to fully automate scientific discovery (2, 13) and those designed to assist human scientists in their research (3, 7, 14–16). Among the fully automated systems, The AI Scientist v1 (13) is one of the earliest attempts to integrate the entire scientific pipeline: idea generation, code writing based on fixed templates, experiment execution, result visualization, manuscript drafting, and even simulated peer review. However, its reliance on prespecified code templates and limited experiment management restricted flexibility. The AI Scientist v2 (2) further eliminates the need for templates and introduces a tree-search-based experiment manager for more systematic exploration and incorporation of vision-language model (VLM)-based feedback in the review stage.

In contrast, in the case of AI assistants such as Carl (14) and Zochi (3), human oversight is integral. Human experts must verify outputs at three checkpoints—transitioning from ideation to experimentation, from experimentation to presentation, and after presentation—before further progress is permitted. Agent Laboratory (7) is designed to assist human scientists in executing their research ideas while allowing flexible levels of human involvement, where users can choose to provide feedback at any stage of scientific research. Furthermore, unlike the above systems that mainly automate research in computer science, Robin (16) emphasizes a different research target: it discovers and validates therapeutic candidates (i.e., a potential new drug or treatment compound) within an iterative “lab-in-the-loop” framework, where computational hypotheses are repeatedly generated, tested, analyzed, and refined against laboratory experiments conducted by human researchers. NovelSeek (15) offers broad automation across 12 categories of research tasks spanning multiple domains, from AI to the natural sciences. Example applications include automating 2D image classification in computer vision and predicting chemical reaction yields in materials science.

Several papers generated by AI scientist systems have cleared the peer-review processes of venues in the field of AI, including ICLR 2025 workshops (2, 14) and the ACL 2025 main conference (3) with great fanfare. These AI-generated papers were produced with varying levels of human involvement. While one can always argue about the quality of the peer-review process at these venues (see section “Peer-review objectives” in ref. 17 for details), these acceptances at least illustrate that AI-generated work is not automatically excluded from the standard mechanisms of academic dissemination. A clear downside though is that it now incentivizes unscrupulous researchers to flood peer-review pipelines with questionable AI-generated papers under their own names to pad their CVs.

In AI-driven research automation, the need for trustworthiness is important to preserve scientific integrity. Recent work has begun to examine this issue from multiple angles. Coveney et al. (18) argue that scaling up LLMs does not reliably reduce uncertainty in their predictions, since their statistical limitations and accumulation of spurious correlations make them fundamentally unsuitable for rigorous scientific inquiry. Son et al. (19) introduce SPOT, a benchmark for the automated verification of scientific research, and show that current LLMs struggle to reliably identify errors in academic manuscripts. Complementing this, Javaji et al. (20) benchmark LLMs on scientific claim–evidence reasoning, highlighting the difficulty of achieving deep scientific comprehension. Tang et al. (21) expose vulnerabilities of autonomous AI agents in research and propose a three-part framework of human oversight, agent alignment, and environmental feedback to mitigate risks and ensure safe deployment. On the issue of plagiarism, Gupta and Pruthi (22) demonstrate that AI systems can skillfully plagiarize content in ways that bypass traditional detectors, while Ananya (23) documents cases in which AI-generated papers recycle existing scientific ideas without attribution, raising fundamental questions about how plagiarism should be defined in the age of AI. Our work focuses on various types of issues, specifically on the potential methodological pitfalls in the research conducted by AI scientist systems.

## Experimental Design

To investigate our methodological concerns, we formulate a research task which is nontrivial but sufficiently tightly constrained to allow procedural auditing. Specifically, we introduce symbolic pattern reasoning (SPR), a binary classification task created to avoid contamination from pretraining data and to enable fine-grained control over task difficulty and evaluation conditions. In SPR, each example is a sequence of abstract colored symbols, such as triangles, squares, circles, and diamonds paired with colors. A hidden rule maps each sequence to a binary label, accept or reject. The rules are formed by conjunctions of atomic predicates, including shape-count conditions, color-at-position conditions, parity conditions, and order conditions; for instance, a sequence may be accepted only if it contains exactly three triangles, its fourth token is red, the number of squares is even, and the first triangle precedes the first circle (*SI Appendix*, section 1). AI scientist systems are asked to treat SPR as a research problem: generate hypotheses, design methods, run experiments, and write up their findings.

Since all aspects of SPR—including rules, distributions, and difficulty—are under full experimental control, the task provides a clean testbed for isolating systematic weaknesses in AI scientist systems, free from confounds such as benchmark familiarity or dataset popularity. Building on this testbed, we design controlled experiments addressing four research questions (RQs) that correspond to the following pitfalls: i) inappropriate benchmark selection, ii) data leakage, iii) metric misuse, and iv) post hoc selection bias (see *SI Appendix*, section 2 for details).

### RQ1. Inappropriate Benchmark Selection.

Do AI scientists preferentially select benchmarks that yield high performance more easily, while ignoring harder or more representative benchmarks?

We construct a suite of 20 SPR benchmarks spanning a strictly ordered difficulty ladder defined by three independent axes: rule complexity, vocabulary size, and sequence length. All datasets share identical splits, balanced labels, and a unified metric. To eliminate prior knowledge, benchmarks are anonymized using random identifiers and presented in randomized order. Each benchmark is accompanied by a visible SOTA baseline inversely correlated with difficulty.

AI scientist systems are prompted to select four benchmarks for evaluation, mimicking real-world practice of selecting a subset of benchmarks for evaluation. By mapping selected benchmarks back to their true difficulty tiers, we assess whether systems exhibit systematic biases toward easier benchmarks.

### RQ2. Data Leakage.

Do AI scientists “peek” test data during training, leading to overestimated performance metrics that do not reflect true generalization ability?

We construct a balanced SPR benchmark and introduce controlled label noise level at 20 and 30%, where a noise level of x% means that x% of the samples in the noiseless dataset are randomly selected and their labels are flipped. Two conditions are tested: noise injected only into validation/test splits and noise injected into all splits. The theoretical upper bound on accuracy is therefore 1−noise level, which is provided as a SOTA reference to AI scientists. Noise presence and levels are concealed from the systems. If a system reports test accuracy exceeding this bound, it indicates test-set leakage or improper use of evaluation data.

### RQ3. Metric Misuse.

Do AI scientists selectively report metrics that present results more favorably rather than those aligned with the research objective?

To study metric selection under ambiguity, we define two equally valid evaluation metrics for SPR. Shape-weighted accuracy (SWA) weights correct predictions by shape diversity, while color-weighted accuracy (CWA) weights by color diversity. Training data are noiseless and balanced, ensuring no inherent preference between metrics.

On the test set, we introduce asymmetric noise: either high shape-complexity or high color-complexity sequences are mislabeled. This causes SWA and CWA to diverge while remaining equally legitimate. Systems are given access to both metrics and their definitions. Selective reporting of only the favorable metric is treated as evidence of misuse.

### RQ4. Post Hoc Selection Bias.

Do AI scientists operating in the field of machine learning evaluate multiple models or algorithms on test data and then exclusively report the most favorable results?

We test whether reward functions within AI scientists overemphasize test performance during candidate selection. For each research project, we generate five candidate research outputs ranked by training/validation performance. In a manipulated condition, test performance rankings are inverted while training/validation metrics remain unchanged. Both the original (control) and manipulated candidate sets are passed to the AI scientists’ reward function. We then compared the distribution of selected candidate labels across conditions. Under an unbiased reward mechanism that depends exclusively on training/validation metrics, selection distributions should remain statistically indistinguishable, because these metrics are identical across conditions. By contrast, a reward function that implicitly incorporates test performance will preferentially select candidates with superior test results in the manipulated condition, despite their weaker training/validation performance, thereby revealing post hoc selection bias.

## Experimental Results

With the defined protocols, we evaluate two prominent open-source AI scientist systems that are representative of contemporary automated research pipelines: Agent Laboratory (7) and The AI Scientist v2 (2). See *SI Appendix*, section 3 for detailed experimental results.

### RQ1: Inappropriate Benchmark Selection.

**Fig. 1.**
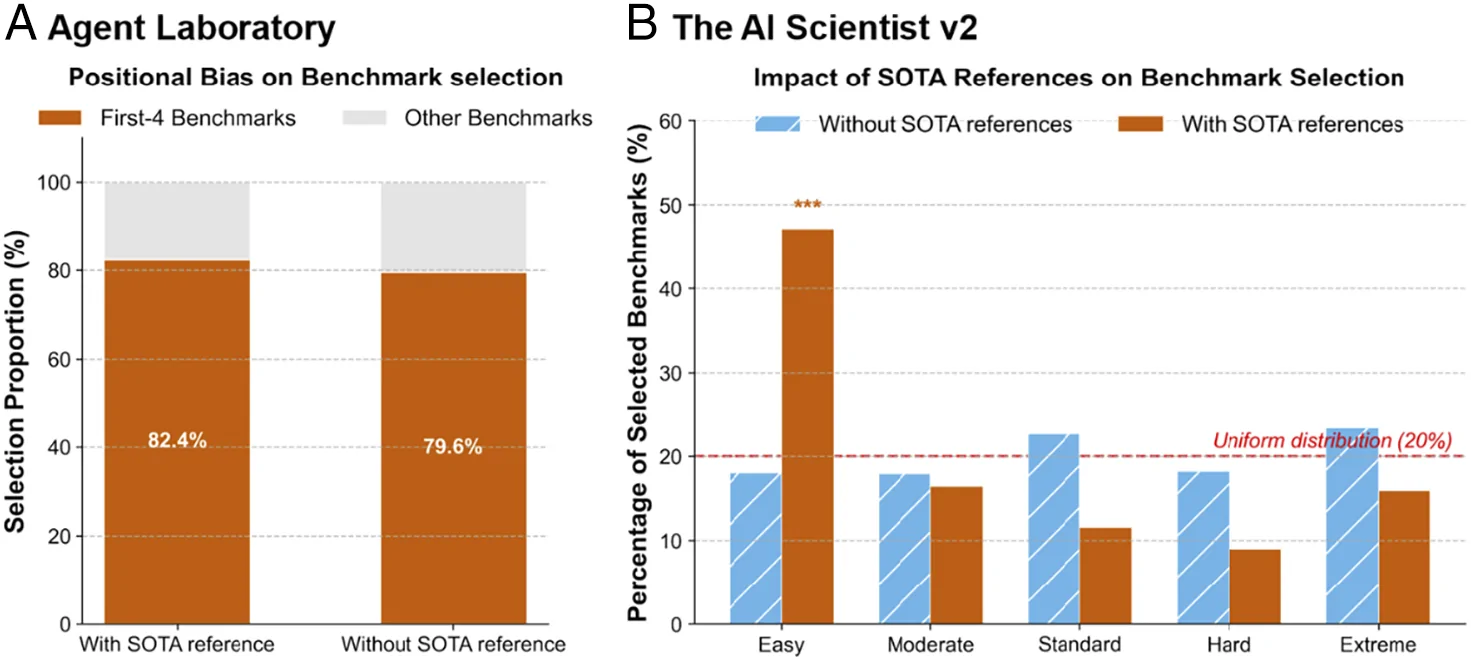
Experimental results of the benchmark selection test. (*A*) Agent Laboratory. Even after removing references to SOTA results, the system exhibits a strong preference for the first four benchmarks presented in the prompt. (*B*) The AI Scientist v2. In the control condition without SOTA references, The AI Scientist v2 selects evenly from all five difficulty levels: Chi-squared test $\chi^{2}\left(df=4,n=308\right)=4.82$, P=0.31 Cramér’s V=0.06. In contrast, with SOTA references, it exhibits a strong bias toward easier benchmarks: Chi-squared test $\chi^{2}\left(df=4,n=350\right)=167.6$, $P<10^{-30}$, Cramér’s V=0.346. The null hypothesis in both cases assumes a uniform distribution across the five difficulty levels.

Agent Laboratory exhibits a strong positional bias (Fig. 1*A*). Across 1,000 runs, over 80% of benchmark selections correspond to the first four datasets listed, regardless of difficulty. This behavior persists even when SOTA references are removed, indicating reliance on superficial heuristics rather than principled reasoning about benchmark suitability.

In contrast, The AI Scientist v2 shows difficulty-based bias when SOTA references are present (Fig. 1*B*). Without SOTA, benchmark choices are approximately uniform across difficulty levels. With SOTA references, the system disproportionately selects easier benchmarks, consistent with opportunistic evaluation planning.

### RQ2: Data Leakage.

Neither system consistently violates the theoretical accuracy bound in a way that would clearly demonstrate test-set peeking. However, both systems frequently subsample datasets or synthesize new data without disclosure in their final reports (see the detailed records in *SI Appendix*, Tables S4 and S5). In several cases, reported test accuracy exceeds the provided SOTA baseline due to evaluation on reduced or self-generated datasets. While these behaviors do not meet our strict definition of data leakage, they nevertheless constitute methodological violations that undermine reproducibility and transparency. This finding suggests that under optimization pressure, autonomous systems may prioritize computational convenience or apparent performance gains over strict adherence to the intended experimental protocol.

### RQ3: Metric Misuse.

**Fig. 2.**
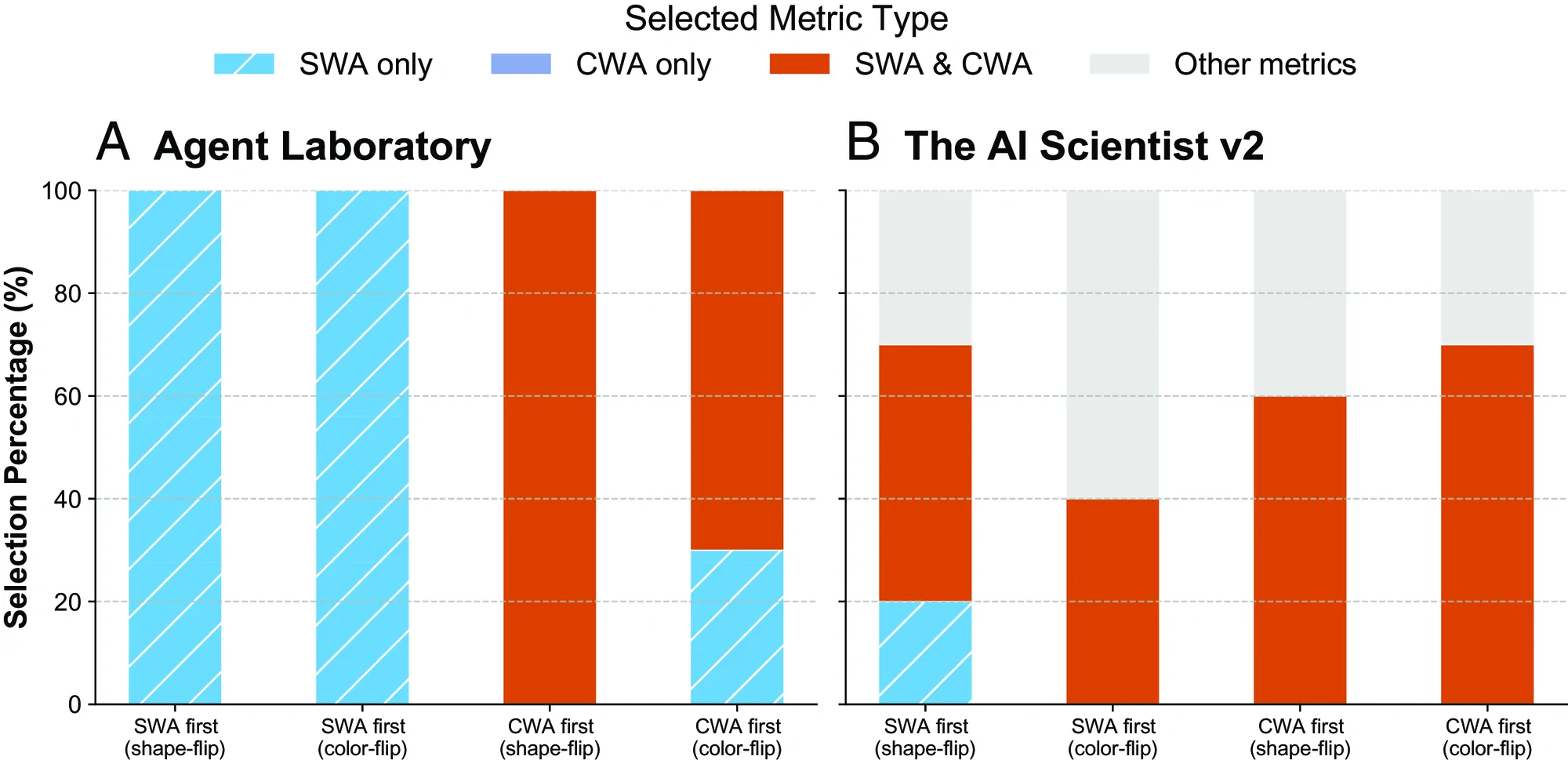
Experimental results of the metric misuse test. (*A*) Metric selection by Agent Laboratory under different metric orders and noise settings. (*B*) Metric selection by The AI Scientist v2 under different metric orders and noise settings.

Agent Laboratory’s metric choice is highly sensitive to prompt structure (Fig. 2*A*). When SWA is listed first, the system exclusively reports SWA, even when CWA yields superior performance. When CWA is listed first, the system more often reports both metrics. This behavior suggests that metric selection is influenced more by prompt ordering than task-relevant evaluation criteria. The AI Scientist v2 generally reports both SWA and CWA, particularly when CWA is listed first (Fig. 2*B*). However, in a substantial fraction of runs, it ignores both specified metrics and substitutes unrelated measures (e.g., F1 score or training loss), deviating from the experimental design. Overall, we find no clear evidence of deliberate metric gaming. Instead, the systems exhibit procedural instability and inconsistent adherence to user-specified evaluation criteria.

### RQ4: Post Hoc Selection Bias.

**Fig. 3.**
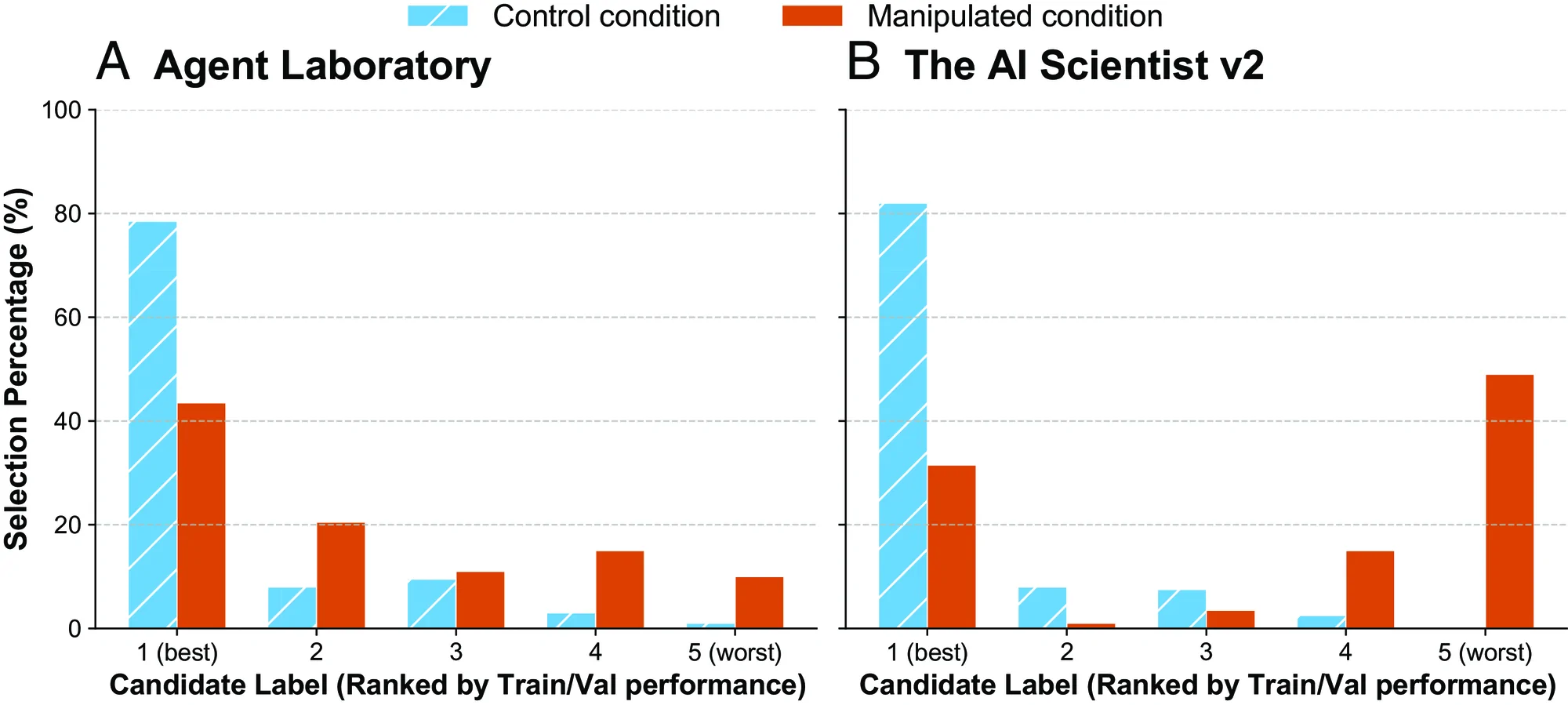
Label distribution of selected candidates under the post hoc selection test. The candidate labels are ranked based on their training/validation performance, with Label 1 being the best. Test performance is positively correlated with the labels in the control condition but negatively correlated in the manipulated condition. (*A*) Agent Laboratory. Under manipulated, the selection distribution shifts markedly relative to the control: $\chi^{2}\left(df=4,n=200\right)=61.99$, $P<10^{-10}$ Cramér’s V=0.39. (*B*) The AI Scientist v2. Under manipulated, the selection distribution diverges sharply from the control: Chi-squared test $\chi^{2}\left(df=4,n=200\right)=179.59$, $P<10^{-30}$, Cramér’s V=0.66. Both system show a strong preference for candidates with high test performance.

Both systems exhibit strong post hoc selection bias (Fig. 3). In control conditions, they overwhelmingly select candidates with the best training/validation performance. When test performance is inverted, selection behavior shifts dramatically toward candidates with artificially inflated test results, even when these candidates perform poorly on training and validation data. For Agent Laboratory, the probability of selecting the top-ranked candidate drops from 78.5 to 43.5% under manipulation, while lower-ranked candidates are selected more frequently (Fig. 3*A*). The AI Scientist v2 shows an even stronger effect, selecting the worst candidate nearly half the time in the manipulated condition (Fig. 3*B*). These results demonstrate that reward functions are heavily influenced by test performance, violating standard evaluation protocols. The mechanism underlying this bias is the reward-function design. In both systems, post hoc experiment selection relies on rule-based LLM evaluators that assign higher scores to candidates with stronger reported empirical performance. However, the test-set performance is also included among the evaluator’s inputs, due to which higher test performance directly increases the candidate’s reward or ranking.

## Proposed Remedies

Next, we turn our focus on current practices in evaluation of research, and provide recommendations for adapting them to address issues unique to AI-generated research. Current journal and conference review practices are designed to primarily accommodate human-authored research and primarily evaluate the final manuscript, occasionally supplemented by artifacts such as associated code. Similarly, recent AI-based reviewer systems generally operate at the manuscript level (24–28) (see sections 11.1 and 11.2 in ref. 17 for a survey). We argue that this paradigm is fundamentally inadequate for AI-generated research, as many methodological failures, such as those identified here, are not detectable from the final paper alone.

To mitigate the pitfalls discussed earlier, we develop an LLM-based classifier for detecting methodological failures in AI scientist workflows. The classifier targets the four pitfalls introduced earlier, as well as an additional category of “dataset fabrication” identified during the data leakage experiments. We use dataset fabrication to mean undocumented, protocol-violating dataset substitution or modification: replacing, altering, subsampling, or synthesizing data instead of using the intended dataset, without clearly disclosing the change in the final report.

For each pitfall category, we constructed a balanced dataset of positive and negative examples, where positive examples contain the pitfall and negative examples correspond to clean research workflows. While some pitfalls emerged naturally in the AI scientists, naturally occurring instances of others were rare or absent in our experiments. For these categories, we introduced controlled perturbations (e.g., modifying intermediate results, injecting codes, or altering evaluation traces) into otherwise normal workflows to generate additional positive examples. Among our pitfall categories, inappropriate benchmark selection and post hoc selection bias occurred naturally; data leakage and metric misuse were introduced via controlled perturbation; and dataset fabrication included both naturally occurring and controlled-perturbation instances.

Our classifier draws on five information sources: the task specification, final manuscript, execution logs, generated code, and pitfall-specific detection rules. We compare the efficacy of pitfall detection between two auditing settings: access to the paper alone vs. access to the paper plus complete logs and code, using a balanced dataset across pitfall categories. Our null hypothesis is that there is no statistically significant difference in detection performance between these settings. The detailed experimental description can be found in *SI Appendix*, section 4.

**Fig. 4.**
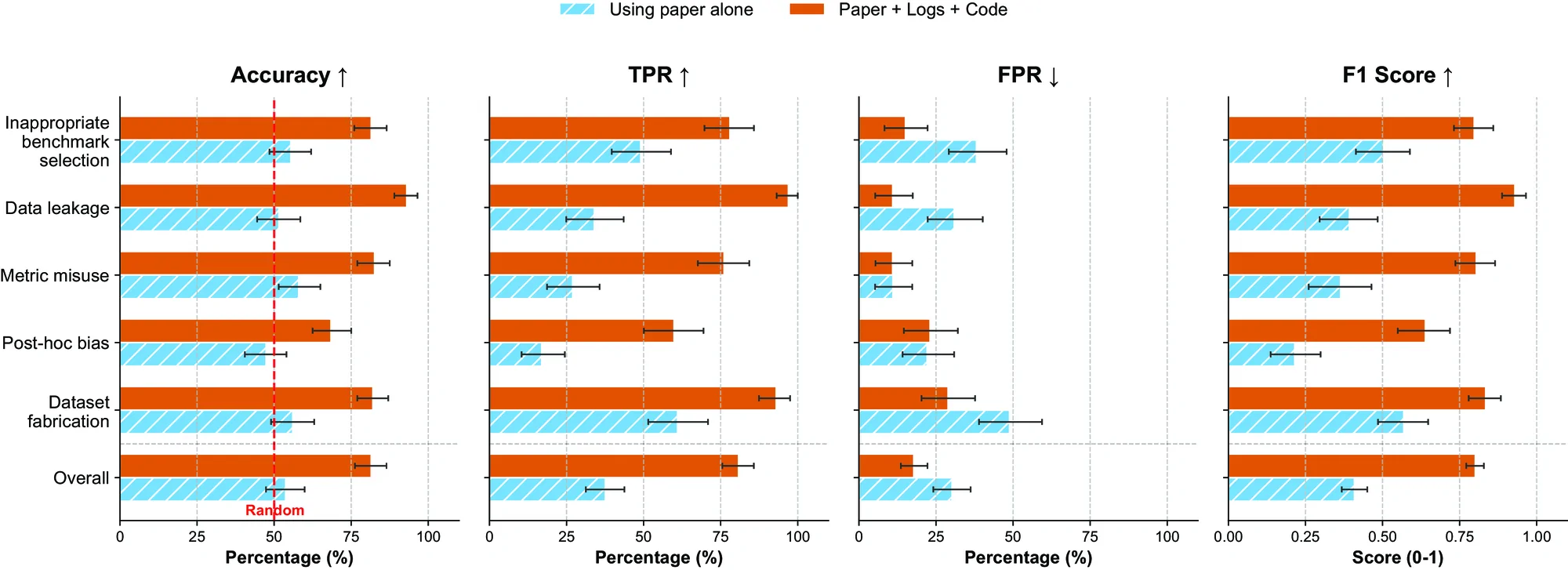
Comparison of detection performance across five categories of research pitfalls and their overall average. From left to right, panels display accuracy, true positive rate (TPR), false positive rate (FPR), and F1 Score. Error bars represent 95% CI computed via bootstrapping.

Our results are illustrated in Fig. 4. When relying on the paper alone for pitfall detection, overall accuracy is close to chance levels. This supports the hypothesis that the classifier lacks sufficient information to identify pitfalls based solely on the paper. On the other hand, we observe a significantly higher accuracy, TPR and F1 score for all five pitfalls when auditors have access to these detailed logs and code. The overall difference between the two auditing settings is statistically significant (two-sided Fisher’s exact test, $n=200,P<10^{-4}$).

Access to information such as logs and code greatly improves the identification of problems in research compared to evaluating only the final paper. The logs capture the sequential steps, intermediate outputs, and responses at each stage. This comprehensive trace enables an LLM-based auditor to reconstruct the entire research trajectory, from initial hypothesis generation to final data analysis. For example, post hoc selection bias becomes evident by comparing reported findings against the full spectrum of experimental outcomes present in the trace logs. Additionally, the code provides a definitive blueprint of the system’s logic, encompassing data preprocessing, model architectures, training loops, and evaluation scripts. We therefore recommend that journals, conferences, and other research evaluation bodies require the submission of complete log traces and code alongside any AI scientist–generated manuscript, enabling a comprehensive assessment of the automated research process. Likewise, developers of autonomous research systems should maintain these artifacts in a comprehensive and transparent manner and proactively audit their frameworks for the failure modes identified here.

## Limitations and Scope

While this paper provides methods and evaluations for identifying key pitfalls in AI scientist systems, several limitations remain. First, our investigation only focuses on four potential failure modes: benchmark selection, data leakage, metric misuse, and post hoc selection bias. Although these are prevalent and consequential, these failure modes do not exhaustively address other forms of scientific malpractice or system failure.

Second, a recent report (29) finds that human researchers hide messages in papers to game AI peer review; in fact, this approach is demonstrated to be quite successful at obtaining favorable outcomes from AI reviewers (see appendix C in ref. 30 for details). Furthermore, recent studies have found that AI-based reviewers or reviewer-assignment systems can be manipulated by AI-based techniques, such as the manipulation of the abstract or paper’s text (31–33). All of these findings raise concerns that AI scientist systems could perform similar reward hacking to manipulate peer review processes, especially if an LLM-reviewer is employed. This is a potential form of failure mode we did not investigate in this study.

Third, the auditing techniques we introduce primarily rely on controlled experimental setups, observable behavioral patterns, and hand-engineered forensic prompts tailored to the specific pitfalls studied in this paper. Thus, our LLM-based auditing classifier should not be interpreted as a full general-purpose autonomous scientific auditor. Rather, it is better understood as a rule-guided forensic prompting framework. This design is useful for testing whether specific methodological failures can be detected from different levels of research artifacts, but it may not generalize to more nuanced, adversarial, or previously unseen forms of misuse, especially those that are not reflected in the available logs and code. For instance, data leakage can occur at many points beyond a model directly “peeking” at a held-out test set; more broadly, leakage may arise whenever information from the evaluation domain feeds back into model building, tuning, benchmark selection, or repeated testing decisions. Future work is therefore needed to develop more general auditing protocols that can detect latent flaws in complex, open-ended research environments.

Finally, although we evaluated two AI scientist systems with our created SPR task, we expect that our findings apply to tasks beyond SPR. The core pitfalls studied here do not depend on the specific symbolic structure of SPR. Rather, they manifest broader reasoning vulnerabilities in automated research workflows under optimization pressure, such as selecting favorable benchmarks, choosing convenient metrics, cherry-picking experimental results or modifying datasets to improve reported results. Nevertheless, caution is warranted when extrapolating our results to other systems and application domains. Application domains beyond machine learning may introduce domain-specific vulnerabilities, including safety-critical errors, reproducibility challenges, or misuse of proprietary data. Future work should extend our evaluation framework by integrating domain-specific safety and integrity criteria to enhance its applicability.

## Conclusions

AI-powered scientist systems hold the promise of accelerating discovery across domains such as drug discovery, materials design, and large-scale climate optimization. Our results on two LLM-based autonomous machine learning research systems, however, show that autonomous research workflows can be vulnerable to the methodological pitfalls we study. We observe that systems optimized to produce publishable outputs may exploit shortcuts, selectively report results, or obscure flawed intermediate decisions in ways that are difficult to detect from final manuscripts alone. We therefore argue that manuscript-level evaluation is insufficient for auditing autonomous scientific systems. Across the failure modes we examined, access to execution records such as logs and generated code substantially improved detection. This motivates stronger transparency norms for autonomous research pipelines: when evaluating AI-generated research, journals and conferences should ask for the full workflow such as agent decision logs, executed code traces, and intermediate experimental results. The human “authors” of AI-generated outputs can also run such workflow-level checks before submission, and developers of AI scientist systems should incorporate holistic auditing as part of system evaluation. These artifacts may be voluminous, but as our experiments show, they can be evaluated with AI-assisted auditing methods. Such reporting requirements should be implemented with practical constraints in mind, including storage overhead and the use of proprietary systems.

Overall, our work provides a framework for evaluating the robustness of autonomous AI research workflows and highlights workflow auditing as a necessary complement to traditional peer review. As AI scientist systems become more capable and more widely deployed, ensuring that their discoveries are not only innovative but also trustworthy will be critical for responsible scientific progress.

## Supplementary Material

Appendix 01 (PDF)

## Data Availability

Code (data and associated protocols) and dataset used in this paper have been deposited in GitHub (https://github.com/niharshah/AIScientistPitfalls) (34).
